# Electronic Coupling and Catalytic Effect on H_2_ Evolution of MoS_2_/Graphene Nanocatalyst

**DOI:** 10.1038/srep06256

**Published:** 2014-09-01

**Authors:** Ting Liao, Ziqi Sun, Chenghua Sun, Shi Xue Dou, Debra J. Searles

**Affiliations:** 1 AIBN Centre for Theoretical and Computational Molecular Science, University of Queensland, Brisbane, QLD 4072, Australia; 2 Institute for Superconducting & Electronic Materials, University of Wollongong, Wollongong, NSW 2500, Australia; 3 School of Chemistry, Monash University, Clayton, VIC 3800, Australia; 4 School of Chemical and Molecular Biosciences, University of Queensland, Brisbane, QLD 4072, Australia

## Abstract

Inorganic nano-graphene hybrid materials that are strongly coupled via chemical bonding usually present superior electrochemical performance. However, how the chemical bond forms and the synergistic catalytic mechanism remain fundamental questions. In this study, the chemical bonding of the MoS_2_ nanolayer supported on vacancy mediated graphene and the hydrogen evolution reaction of this nanocatalyst system were investigated. An obvious reduction of the metallic state of the MoS_2_ nanolayer is noticed as electrons are transferred to form a strong contact with the reduced graphene support. The missing metallic state associated with the unsaturated atoms at the peripheral sites in turn modifies the hydrogen evolution activity. The easiest evolution path is from the Mo edge sites, with the presence of the graphene resulting in a decrease in the energy barrier from 0.17 to 0.11 eV. Evolution of H_2_ from the S edge becomes more difficult due to an increase in the energy barrier from 0.43 to 0.84 eV. The clarification of the chemical bonding and catalytic mechanisms for hydrogen evolution using this strongly coupled MoS_2_/graphene nanocatalyst provide a valuable source of reference and motivation for further investigation for improved hydrogen evolution using chemically active nanocoupled systems.

Sustainable hydrogen production has attracted growing attention, and an advanced catalyst for the electrochemical hydrogen evolution reaction (HER) that results in increased efficiency of this important electrochemical process is urgently required^1^,^2^,^3^. Recent work showed that the transition-metal dichalcogenide semiconductor MoS_2_ nanocatalyst, which basically consists of two-dimensional S-Mo-S layers (or ‘sandwiches') that are stacked to various degrees, is a promising electrocatalyst for the HER^4^,^5^,^6^,^7^,^8^,^9^,^10^. The catalytic reactivity of the S-Mo-S layers is associated with their edges while their basal planes were catalytically inert. Nanosized MoS_2_ with exposed edges should be more active for HER electrocatalysis than materials in bulk forms^11^,^12^,^13^. Hence, the formation of nanoscale contact between MoS_2_ and another electronically dissimilar material will open up new routes toward novel semiconductor based devices using the advanced catalytic activity of this reduced dimensional material.

Graphene, among all kinds of carbon materials, seems to be particularly attractive due to its unique two-dimensional honeycomb structure that leads to unusual electronic and mechanical properties and may provide an ideal support for semiconductor nanocatalysts^14^,^15^,^16^,^17^. Depending on the reaction considered, the role of the graphene support may be limited to the stabilization of small particles that contain atoms with low coordination numbers, or may help to stabilize or activate some of the reactants on the semiconductor itself. Indeed, nanolayers of the semiconductor MoS_2_ supported on reduced graphene oxide sheets have shown advanced electrocatalytic and photoelectrochemical properties in the production of H_2_^16^,^17^. It was found that there is a positive synergetic effect between MoS_2_ and the graphene oxide support, enabling the H_2_ to be easily evolved from the supported MoS_2_ nanolayers^16^. Unlike conventional catalysts that have larger sizes, the catalytic performance of semiconductor nanolayers is often controlled by quantum size effects owing to their highly reduced dimensions, and is therefore sensitively dependent on the properties of atoms that have lower coordination numbers than they would typically have in the bulk. A clear understanding of the nature of the influence of the support on the catalytic properties of the supported semiconductor nanolayers using theoretical computation may provide insight that leads to new options for nanoscale catalysts.

A complete picture of the kinetics of the hydrogen evolution reaction (HER) involves, firstly, a primary discharge step (Volmer reaction) where bonding of hydrogen to the catalyst H^+^ + e^−^ + * → H* occurs (* denotes an active site on the catalysis surface able to bind to hydrogen), followed by either an electrochemical desorption step (Heyrovsky reaction) or a recombination step (Tafel reaction) H* + H* → H_2_. To date, the reaction mechanisms on MoS_2_ were inconclusive because of the wide range of HER Tafel slopes reported^18^,^19^. The process involving hydrogen bonding to the catalyst and the recombination reaction is more inherently correlated with the catalyst itself and is an essential step of the HER. Knowledge of the H* bonding structure and the reactivity of the recombination process on the nanolayered MoS_2_ catalyst supported on graphene sheet is therefore of fundamental importance for understanding the reaction that takes place during HER. The two types of exposed edges which both have predominantly unsaturated atoms, may provide different bonding geometries with hydrogen, resulting in a dual functionality for the hydrogen evolution reaction. Besides edges, the kink sites that exist as joints between the Mo and S edges may also have some effect on the overall catalytic activity. The presence of corners and kink sites was revealed by experimental studies, but the role of kink sites has not previously been considered^20^. The mechanism and reaction pathways of the HER with MoS_2_ catalysts without support also remained inconclusive. Detailed information about the peripheral structures of MoS_2_ of both free-standing nanolayers and when supported on a graphene sheet is thus essential in order to understand the nature of their catalytic activities.

In this paper, we analyze from a computational point of view the chemical and electronic coupling mechanism between the MoS_2_ nanolayer and vacancy mediated graphene support. In addition, the nature of the active sites of the MoS_2_/graphene nanocontact involved in the essential step of the hydrogen evolution reaction (HER), that is, hydrogen bonding and recombination, was studied. The functionality of the MoS_2_ nanolayer when a graphene base is present turns out to be different for the two types of edges, wherein the Mo edge becomes more active in facilitating the hydrogen evolution reaction with a lower hydrogen binding energy |Δ*E*_bond_| and hydrogen recombination barrier compared with the free-standing MoS_2_ nanolayer, but the catalytic effect of support on the S edge is the opposite. The calculated results provide a motivation for further investigations on improved hydrogen evolution using supported MoS_2_ and provide a valuable source of reference of the use of the chemically active nanocoupled systems as well.

## Results

**Figure 1:**
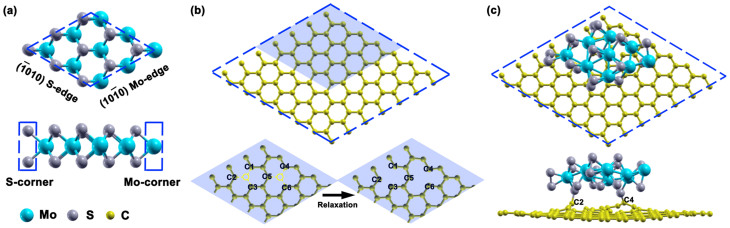
(a) Two views of the free-standing 3 × 3 × 1 MoS_2_ nanolayer before structural optimization. (b) The top image shows the 7 × 7 × 1 graphene supercell with MoS_2_ nanolayer sitting area highlighted as a blue shadow, and on the bottom image the atomic structures of the reduced graphene in the shadow area before and after structural relaxation. (c) The optimized structure of the MoS_2_/graphene nanocontact. The cyan, grey and yellow spheres represent Mo, S, and C atoms, respectively. The corner sites are highlighted by dashed rectangles in (a). The missing carbon atoms are highlighted as yellow circles and the neighboring carbon atoms with dangling bonds are denoted with numbers in (b).

MoS_2_ consists of layers of S-Mo-S sandwiches. The stoichiometry and coordination numbers of the edge atoms can differ from the bulk. The most active sites are Mo edges passivated by S atoms that have low coordination numbers, providing a sulfide rich environment^19^. Although the S-saturation of the Mo edge observed in experiments is mostly S-rich, by altering the external environment, such as exposure to atomic hydrogen, the S-saturated Mo edge can be reduced to pure Mo edge again with missing sulphur atoms^21^. The binding energies of sulphur atoms to the Mo edges of MoS_2_/graphene nanocontact and free-standing MoS_2_ nanolayer were compared, and as shown in Supplementary Table 1, the binding energies of sulphur atoms at the Mo edge decrease almost 30% when the reduced graphene support is present. Therefore the defective graphene support stabilises a MoS_2_ nanolayer which is deficient in S. Therefore, in this manuscript we focus on the case where a MoS_2_ nanolayer with pristine edges is allowed to relax to its minimum energy structure since this is what occurs under standard reaction conditions and is therefore an environment that often occurs experimentally. Figure 1a displays the two kinds of bare edge of the MoS_2_ nanolayer, the sulphur-terminated 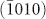 edge and the molybdenum-terminated 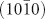 edge, as well as the active S- and Mo-corner sites which are highlighted in the dashed boxes. This is motivated by the frequent observation of steps and corners in nanosized MoS_2_ using high-resolution TEM telescope^20^. To produce a vacancy mediated graphene sheet, two carbon atoms were removed from the graphene sheet, directly beneath the MoS_2_ nanolayer, and the structure was optimised. This leads to the presence of a number of dangling bonds on the neighboring carbon sites, as denoted with numbers in Figure 1b, which can efficiently chemically interact with the MoS_2_ nanolayer. Figure 1c displays the optimized structure of MoS_2_ on the vacancy mediated graphene surface showing the covalent bond that is formed between them, i.e. between the dangling bond of the graphene surface (C2 and C4 carbon atoms) and the edge sulphur atom of the MoS_2_ nanolayer. These S-C bonding distances are ~(1.76–1.78) Å, consistent with typical bond lengths in most sulphur compounds. The real applications of nanocatalysts supported on graphene are limited as weak bonding is usually found, involving the π orbital perpendicular to the graphene plane^22^. On one hand, the underlying graphene can hardly modify the reactivity of supported nanoparticles due to this weak interaction. On the other hand, the anchored nanoparticles are highly mobile on graphene due to the weak interaction and in some cases they easily agglomerate into large particles. A common practice in heterogeneous catalysis, controlling the catalytic performance via tuning the interaction between the reactive nanoparticles and the underlying support, is to introduce sp^2^ dangling bonds on the graphene surface as a result of the formation of carbon vacancies, resulting in vacancy-mediated graphene^23^,^24^. Most importantly, strongly coupled inorganic nano-graphene hybrid materials stabilized via this sort of chemical bonding usually present superior electrochemical performance than the traditional weakly contacted counterparts.

We found that the optimized structure of the MoS_2_ is quite similar whether or not the support is present, and therefore its optimised geometric shape in isolated form is not displayed. The optimized structure in the presence of the support is shown in Figure 1b. The MoS_2_ nanolayer undergoes considerable deformation from the bulk structure on structural optimization. As is clear from Figure 1b, the atomic displacements of the inner S atoms on the 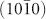 Mo edge sites during the geometry optimization are significant with extreme outward relaxation to the position even beyond the outmost metallic Mo neighbors when projected along the z axis. The interacting bonds of these displaced S atoms to the inner metallic neighbors is either elongated or broken. In contrast, the atomic displacements on 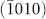 S edge are small. Hence, the optimized outcomes for each edge, Mo- or S-, turn out to be similar to each other with sulfide atoms in the outmost exposed positions, which make them the most likely to be involved in reaction with hydrogen atoms when they are exposed to them. After optimization, the Mo-corner is characterized by two more Mo-Mo bonds and results in a four-coordinated Mo atom, as shown in Figure 1b. Two S atoms appear in the optimized configuration of the S-corner, which are bound to each other and form a disulfide linkage triangular unit, which has been reported as a stable MoS_2_ edge configuration in sulfide rich environments based on thermodynamic calculation and TEM observation, i.e.^19^.

**Figure 2:**
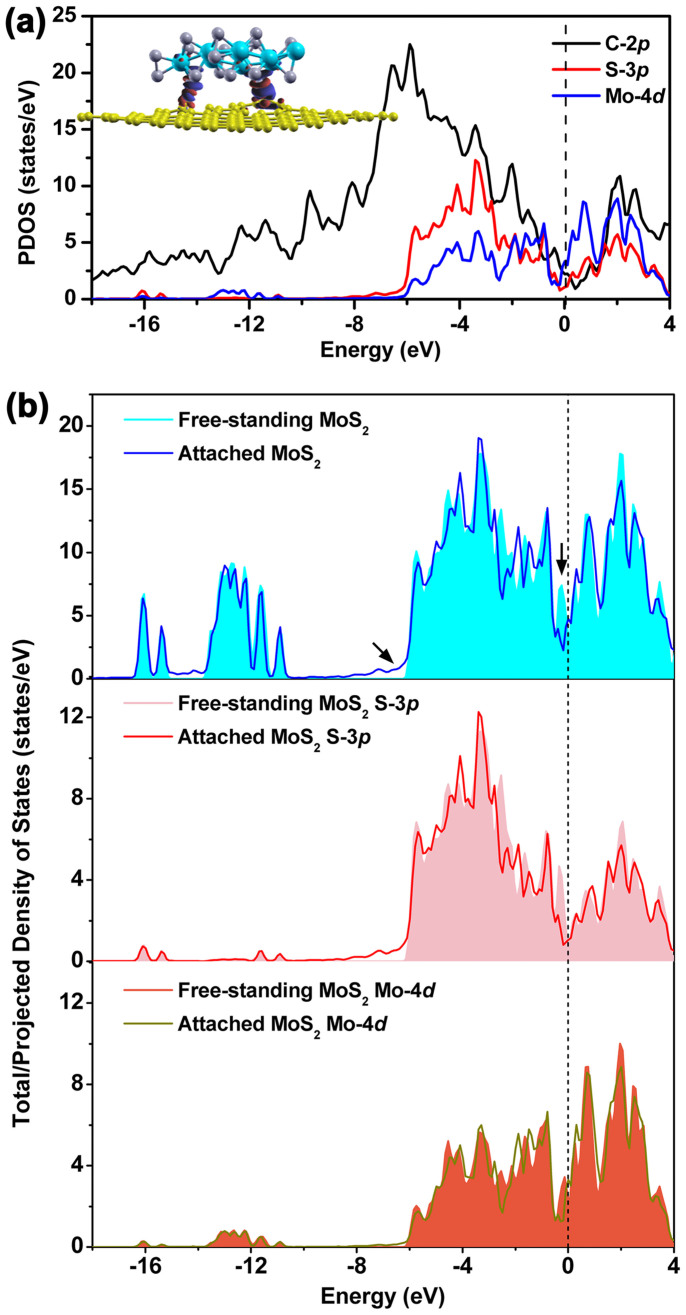
(a) Orbital-decomposed partial density of states for each atom in nanocontacting model. The isosurface of the electron charge density difference with the isovalue of 5.0 × 10^−3^ |e|Å^−3^ is plotted in the inset with the red region denoting the electron gain and the blue region the electron loss. (b) Total and projected density of states (TDOS/PDOS) of MoS_2_ nanolayer attached on graphene sheet and as the free-standing one as well. The distinctive changes are pointed out by the arrows in the top part.

Characterization of the electronic bonding between the MoS_2_ nanolayer and graphene support was carried out. As shown in Figure 2a, there are several energy levels from −8.0 eV until the Fermi level aligned at the same positions in the projected density of states (PDOS) of MoS_2_/graphene, and significant overlap (i.e., orbital hybridization) was found between 3p orbitals of S and 2p orbitals of C in the wave functions, also indicating a strong covalent bond is formed between the nanolayer and the support. The character of the charge redistribution between the MoS_2_ nanolayer and graphene sheet can be also evaluated as the difference between the electron charges of the MoS_2_ nanolayer and graphene sheet alone and the one formed when they are in contact, and is shown in the inset of Figure 2a. The red colour denotes gain of electrons and the blue colour denotes electron loss. The contact-induced major charge redistribution is not equally distributed among all atoms but mainly occurs in the contact region between the MoS_2_ nanolayer and graphene support, originating from the vacancy-mediated hybridization between the C 2p and S 3p orbitals.

Next, we compare the total and projected density of states for two cases: the free-standing MoS_2_ nanolayer and the one in nanocontact with the graphene support, shown in Figure 2b. From these results, one can determine the trend of the charge flow in the MoS_2_ layer as a whole. In both cases, the periphery-induced relaxation breaks the symmetry of semiconducting bulk MoS_2_, leading to the presence of metallic states at the Fermi energy level. Note that the states of the attached MoS_2_ nanolayer are similar to that of the free-standing one, except for two distinctive changes as pointed out by the arrows in the top part of Figure 2b: one is the obvious diminishing of the metallic electronic states around the Fermi energy level which are dominantly derived from the unsaturated S atoms at the periphery sites; the other change is highlighted by the arrow around −8.0 ~ −6.0 eV indicating occupied states are present when the MoS_2_ is bound to the graphene substrate. Based on the analysis of the projected density of states to the atomic sites as shown in Figure 2a, the extra state in this lower energy level is associated with the bonding characteristic of the C atoms in the support in the attached case.

It has been unequivocally demonstrated that the rate of H_2_ formation on MoS_2_ catalysts directly correlates with the mean MoS_2_ particle diameter^16^, suggesting that the active sites for H bonding are located at the periphery around the MoS_2_ particle, which would correspond to atoms in corner or edge positions in nanolayers above the support interface. To find out whether the decreased metallic nature of the states associated with the active periphery sites of the MoS_2_ nanolayer in the supported case affects their catalytic activity in hydrogen evolution, we first probed the chemical bonding of hydrogen atoms in the free-standing nanoparticle using the supercell model. In the peripheral sites (edges and corners) the cationic Mo atoms and anionic S atoms usually have low coordination numbers. Both of these are able to coordinate H, and therefore, they are active for initial bonding with H atoms. Our calculations identified the S and Mo atoms in the supercell which have low coordination numbers as potential active sites on which H atoms can be bonded and recombine (which were located at the periphery of the MoS_2_ layer). Binding energies of single or two H atoms on a free-standing or supported MoS_2_ nanolayer were calculated as 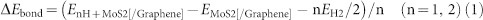
with *E*_nH-MoS2[/Graphene]_ being the total energy of the hydrogen bonded to the complex with or without the graphene support, *E*_MoS2[/Graphene]_ being the total energy of the corresponding catalyst model, and *E*_H2_, the energy of the H_2_ molecule placed in a 10 × 10 × 10 Å^3^ cubic box.

A general principle of catalysis, the Sabatier Principle, is that optimal catalytic activity will be achieved on a catalytic surface which has intermediate binding energies (or free energies of adsorption) with the reactive intermediates^25^. It is unlikely that the intermediate will be activated by a weak bonding with the catalytic surface, and it will be easily contaminated if there is a strong bonding with the intermediate. So a compromise between these two extremes with an intermediate binding energy is key to an efficient catalytic reaction. In the particular case of hydrogen evolution, past studies have demonstrated that this can be quantified by considering the free energy of hydrogen bonding Δ*G*_bond_, and this has been found to serve as a reasonable descriptor of hydrogen evolution activity for a wide variety of metals and alloys^26^,^27^,^28^. The optimum hydrogen binding free energy Δ*G*_bond_ should be around 0. The phonon contribution to the free energy is typically quite small, <5%, which is negligible in the supercell model used. Ignoring entropy terms we can set |Δ*G*_bond_| approximately equal to enthalpy |Δ*E*_bond_| and we therefore use calculated value |Δ*E*_bond_| to judge the catalytic activity for a variety of peripheral sites of MoS_2_ based catalysts, and we will assume in the following that the closer |Δ*E*_bond_| is to zero, the better the catalytic activity of specified periphery sites. The free energy of hydrogen bonded states Δ*G*_bond_ was also estimated using the approximated method proposed by Nørskov by simplifying the entropy energy difference to 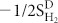, where 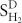 is the entropy of H_2_ in the gas phase at standard condition, for isolated MoS_2_ nanolayer and MoS_2_@graphene as well (Supplementary Figure 1)^27^. However, these differences of the hydrogen binding energies Δ*G*_bond_ at different edges hardly change the predicted relative activities for them by using Δ*E*_bond_.

**Figure 3:**
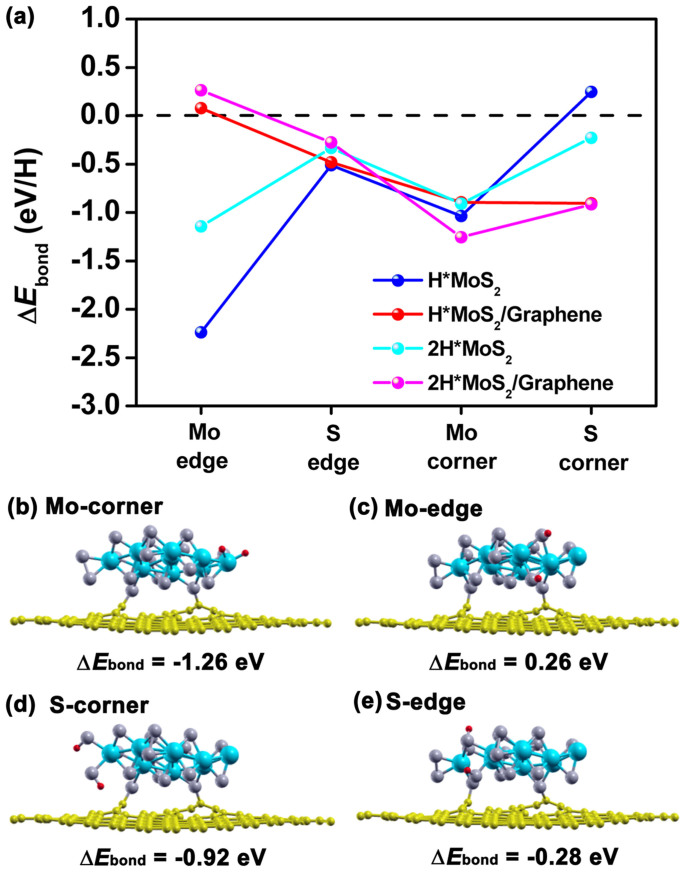
(a) The calculated Δ*E*_bond_ (H and 2H) at the peripheral sites of MoS_2_/graphene nanocontact and free-standing MoS_2_ nanolayer, and (b) the optimized structures of the reactants 2H. The H atoms are red.

In Figure 3a, we have plotted the calculated values of Δ*E*_bond_ when hydrogen is bound to the peripheral atoms of the isolated MoS_2_ nanolayer and MoS_2_ in contact with the graphene substrate and show structural configurations for the latter (H bonded isolated MoS_2_ layers show similar structures to the attached one so are not displayed). We found that for the free-standing MoS_2_ nanolayer, H can bind to the S- or Mo-edge and Mo-corner, resulting in negative Δ*E*_bond_, as shown in Figure 3a. The H atoms bound to the S-atom exposed Mo-edge systems yield very stable structures in which the two H atoms are bound to different S atoms at different levels of the MoS_2_ distorted sandwich structure. The S-edge less strongly binds the H atoms with a |Δ*E*_bond_| which is closer to zero, suggesting for isolated MoS_2_ particles, the S edge could play a major role on its catalytic performance for hydrogen evolution. A stable complex of H atoms bound on the Mo corner is formed in which the two H atoms are bound to the same corner Mo atom. The main difference between the isolated and attached MoS_2_ nanolayers is that the atomic sites at the Mo-edge have a positive Δ*E*_bond_ in the graphene attached MoS_2_, with a much lower magnitude than that obtained for the isolated MoS_2_ nanolayer. For free-standing MoS_2_, the S-edge, with its low H binding energy, is the predominant factor which would lead to a high activity for the HER of the whole layer. The reactivity of the graphene supported MoS_2_ nanolayer might be a better catalyst for the HER because the Mo-edge can contribute to its catalytic activity with a much reduced |Δ*E*_bond_|. Although in our study the support does not play any direct role in bonding or activating the H atoms, except for the role of stabilizing and modifying the MoS_2_ nanolayer, the S atoms on the Mo-edge which have low coordination numbers will be less trapping of the H atoms. This could be the result of the fact that some metallic states associated with the unsaturated peripheral sites are missing after the MoS_2_ is bound to the reduced graphene support, and therefore the MoS_2_ will more easily release H in the next step of electrochemical hydrogen evolution.

The effect of inclusion of a van der Waals correction on the conclusions was considered for the two-hydrogen-atom binding energy at the Mo and S edges of MoS_2_/graphene nanocontact and free-standing MoS_2_ nanolayer. As shown in supplementary Table 2, the introduction of vdW correction alters the absolute value of calculated binding energy less than 0.05 eV at the Mo edge in both cases, yet leaves the binding energies almost unchanged at the S edge. Hence the order of the relative stability of hydrogen bonded each edge is preserved when the vdW correction is taken into account.

Among the different possible MoS_2_/graphene structures with two hydrogens, we also selected the edge sites that weakly bind hydrogen and the corner sites that strongly trap hydrogen and have calculated the complete reaction path for H recombination and release after two atoms are attached to the substrate. The initial state was selected to be those shown in Figure 3b–e, and the final state was selected to be the local minimum energy configuration that a hydrogen molecule placed near the edge or corner would adopt. Activation energies *E*_act_ were then obtained as the energy difference between the maximum energy transition state and the initial adsorption complex along the reaction pathway, and reaction energies Δ*E* were obtained as the energy difference between initial and the final state, and these results are shown in Table 1.

**Figure 4:**
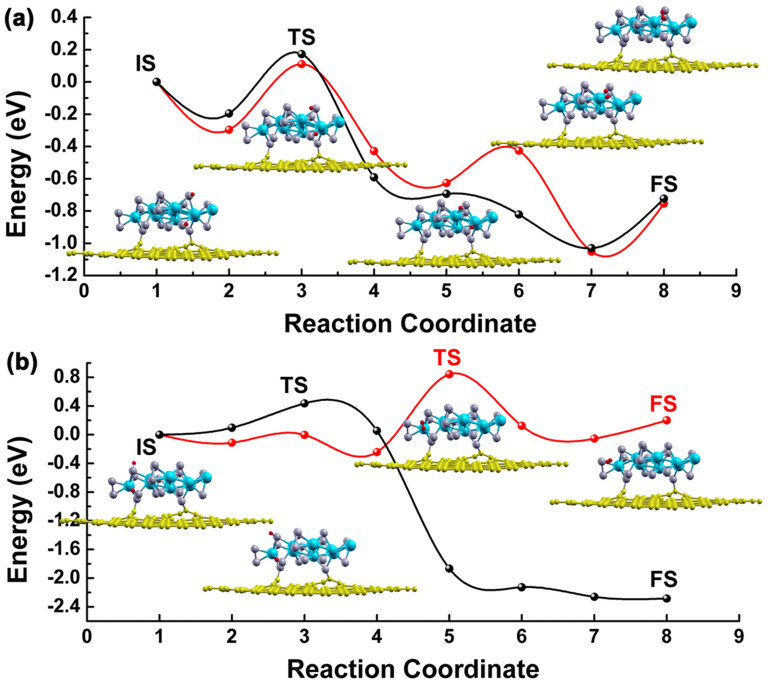
Calculated energy profile involved in the recombination of 2H on (a) Mo-edge and (b) S-edge of MoS_2_ nanolayer as supported on graphene sheet (red curve) and isolated (black curve) for comparison. The optimized structures of selective images along the energy path for MoS_2_/graphene nanocontact are also plotted in the inset. The curves serve to guide the eye.

In Figure 4, we show the minimum energy profile along the H recombination path at the a) Mo and b) S edge sites for the isolated and attached MoS_2_ nanolayer, relative to the energy of the initial structure in each case. The detailed atomic configurations of the initial state, a number of intermediate states, and the final state are also plotted. For two H atoms on the Mo-edge, the global process is exothermic with an activation barrier around 0.17 eV for the free-standing MoS_2_ nanolayer and 0.11 eV for the attached nanolayer. During the reaction path the first step is a rotation of one H atom on the S site followed by a later migration to a high coordination number inner Mo atom of the nanolayer. After overcoming an energy barrier in the subsequent step, we found both H atoms are situated above the nanolayer and come closer to each other. The distance between them decreases to 0.75 Å in the final state, indicating that an H-H bond is formed. The lower barrier of H recombination from the Mo edge on attached MoS_2_ could be due to the nanocontact-induced diminishment of metallic states on filling the antibonding 2π* orbital of formed H_2_. We note that the final energy of the H_2_ on the substrate is lower than the separated substrate and H_2_, which might suggest that it remains bound at low temperatures.

The first stage of the recombination and release of H_2_ from the S edge might be qualitatively considered to be similar to that on the Mo edge but the energetics differ. The energy barrier was determined to be 0.43 and 0.84 eV, respectively, as the H atom is released from the S of the free-standing and attached MoS_2_ nanolayers. For the isolated MoS_2_ nanolayer, rearrangement to form H_2_ results in an even more stable structure, indicating that this site will not be useful for the HER at moderate temperatures. For the MoS_2_/graphene, there is only a small difference in energy between the initial and final structures as the H_2_ is formed, however the activation energy is significant, making this process difficult to proceed. Therefore, the evolution of H_2_ from the S edge becomes more difficult due to the increase in the energy barrier. Nevertheless in the presence of the graphene, H_2_ evolution is enhanced due to the easier evolution path on the Mo edge sites, resulting from a decrease in that energy barrier. The excellent HER activity of the MoS_2_/RGO hybrid catalyst reported by Li et al.^16^ can be understood as Mo edge sites are the active sites mainly involved in boosting the hydrogen evolution whereas the S edge remains inactive.

**Figure 5:**
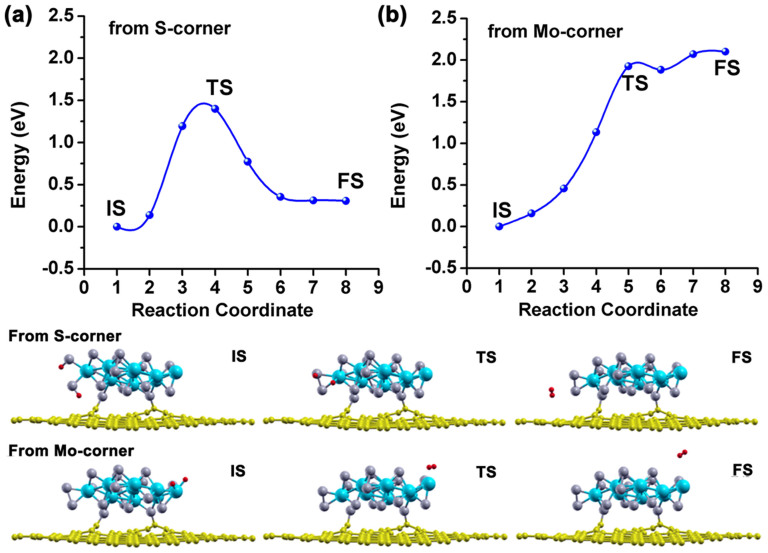
Calculated energy profile involved in the recombination of 2H on (a) S-corner and (b) Mo-corner of MoS_2_/Graphene nanocontact. The optimized structures of initial, transition, and final structures along the paths are also plotted. The curves serve to guide the eye.

The activation energies for the recombination and release of hydrogen were determined to be 1.40 and 2.10 eV, respectively, for highly trapped S and Mo corners in the case of MoS_2_/graphene substrate. The hydrogen atoms at the S corner stay bound to different S atomic sites initially. As shown in Figure 5, when they start to react, one of the H atoms remains bonded to the S corner site while the other migrates to a nearby Mo neighbour. The subsequent reaction process involves both H atoms breaking from the bonded sites and the H_2_ molecule is assumed to form, but has slightly higher energy than the initial structure. From the Mo-corner site, the two H atoms are initially bound to the same corner Mo atom. The meta-stable product on the potential energy surface, that is 0.22 eV more stable than the physically bonded H_2_, consists of two H atoms located on bridge positions near the Mo corner site, with H-H optimized distances around 0.78 Å. Along the further reaction pathway, the H–H bond length starts to decrease to a final equilibrium value of 0.75 Å.

## Discussion

In order to understand and improve the H_2_ evolution process on a MoS_2_ nanolayer without reducing its high activity, we have investigated by means of DFT calculations the nature of the active periphery sites involved in the H bonding and later recombination and release of hydrogen on a series of models in which a graphene support is in effect. We found that the coupling with the reduced graphene support causes noticeable changes in the shape of the Mo-edge in MoS_2_ nanolayer and also a certain degree of charge transfer. The optimized Mo-terminated 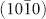 edge in the MoS_2_ nanolayer turns out to be similar to the S-terminated 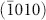 edge with inner S atoms relaxing outward to positions that most readily with the hydrogen atoms, if in contact with them. The charge redistribution that results mainly occurs in the contact region between the MoS_2_ nanolayer and graphene support, originating from the vacancy-mediated hybridization between the C 2p and S 3p orbitals. When MoS_2_ is attached to graphene, the metallic states around the Fermi energy level in association with the unsaturated S atoms at the periphery sites in MoS_2_ also decrease as electron transferring to form contact path with reduced graphene support.

We also checked all of the possible active sites on the MoS_2_/graphene nanocontact using supercell methods that have been shown to be suitable for this type of system. Considering the favourable peripheral sites, the hydrogen binding energies were used to evaluate the catalytic activity, and the activation energy for the essential hydrogen evolution step of the recombination process was determined. The Mo edge of MoS_2_ nanolayer in the presence of graphene support clearly bound the H atoms less strongly, as was revealed by the small |Δ*E*_bond_|, which is enough to modify the energy profile so that the following H recombination and release process becomes relatively easier with a decreasing activation energy to 0.11 eV. In contrast, the same H evolution on S edge becomes more difficult due to a higher energy barrier of 0.84 eV for the MoS_2_/graphene. The functionality of MoS_2_ nanolayer when a graphene base is present is different depending on the types of edges, wherein an improved synergistic catalytic effect was obtained on Mo edges characterized by lower hydrogen binding energies |Δ*E*_bond_| and hydrogen recombination barrier, but an opposite catalytic effect of graphene support was found on S edges.

## Methods

Here we consider a supercell model of MoS_2_ having a 3 × 3 in-plane periodicity and a single layer on the z axis of the unit cell, see Figure 1a (note that the structure of the isolated nanoparticle is not optimized in this figure). This MoS_2_ model system was studied as an isolated structure and also when supported on a 7 × 7 × 1 graphene basal plane repeated in a supercell geometry and with two carbon atoms removed (see the resulting optimized geometry in Figure 1b). The stacking pattern is not considered here as for an adatom or highly anisotropic adsorbant it will be of essential importance, but its effect is small for large adsorbants especially of high symmetry. For a better comparison, the calculations of the isolated MoS_2_ nanolayer were done using the same supercell employed for the MoS_2_/graphene systems. A vacuum region larger than 15 Å between vertically repeated models was used, which is large enough to avoid interaction between the periodically repeated layers or between the one with bonded hydrogen. The MoS_2_ nanolayer considered has a typical crystalline hexagonal shape with the most reactive (1010) facet exposed for both the Mo-terminated 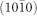 edge and the S-terminated 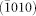 edges. Unless otherwise stated, the position of all atoms was always fully relaxed. The bonding of the hydrogen atoms to both the isolated and graphene-supported MoS_2_ nanolayers was examined.

All calculations were based on density functional theory (DFT) as implemented in the PWSCF code^29^. The spin-polarized generalized gradient approximation (GGA) in Perdew-Burke-Ernzerhof (PBE) format was included^30^. The Kohn-Sham orbitals used to obtain the electron density were expanded in a plane wave basis set with a kinetic energy cutoff of 40 Ryd, and the effect of the core electrons was taken into account by means of the Ultrasoft Pseudopotential (USPP) method^31^. Given the large size of the supercells employed, calculations were carried out at the Γ k-point of the Brillouin zone. The validity of only adopting a Γ k-point to describe our supercell model was tested using denser k grids of 2 × 2 × 1 and 4 × 4 × 1. The difference in the calculated adsorption energy of single hydrogen atom on the Mo edge site of MoS_2_/graphene is smaller than 0.002 eV, which validates the suitability of our current computational methodology. In optimizing the atomic structure, a conjugate-gradient algorithm was employed and the force convergence criterion was set to 0.01 eV/Å.

After the evaluation of the active bonding sites for H atoms and its probable recombination path, the next step was to find the activation energy along that path, so that kinetics of the recombination reaction could be predicted. The climbing-image nudged elastic band (CI-NEB) method, widely used for locating transition states, was adopted to find the minimum energy path (MEP) between a given initial and final state of the recombination reaction^32^. An initial path was constructed and represented by a discrete set of images of the system connecting the initial and final states. Adjacent images were connected by springs, mimicking an elastic band and the tangent of the path is estimated on each image. An optimization of the band, mainly the minimization of the forces acting on images, brings the band to the MEP, through which the highest-energy configuration climbs uphill to the saddle point.

## Author Contributions

T.L. performed the calculations, analyzed the results and wrote the manuscript. Z.Q.S. conceived the idea and reviewed the manuscript. C.H.S. and S.X.D. took part in discussion. D.J.S. discussed and reviewed this manuscript.

## Supplementary Material

Supplementary Information Dataset 1

## Figures and Tables

**Table 1:** The calculated activation *E*_act_ and reaction Δ*E* energy (in eV) for the hydrogen recombination process on the edge and corner sites of the MoS_2_/graphene nanocontact and free-standing MoS_2_ nanolayer. Activation energy *E*_act_ is the energy difference between the maximum energy transition state and the initial adsorption complex along the reaction pathway, and reaction energy Δ*E* is the energy difference between initial and the final state

*E*_act_/Δ*E*(eV)	MoS_2_/Graphene	MoS_2_
Mo-edge	0.11/−0.75	0.17/−0.72
S-edge	0.84/0.20	0.43/−2.2
Mo-corner	2.10/2.10	
S-corner	1.40/0.31	
