# Supplementary material for: Electronic Coupling and Catalytic Effect on H2 Evolution of MoS2/Graphene Nanocatalyst
Source: Sci Rep. 2014 Sep 1;4:6256. doi: 10.1038/srep06256 (PMC4150123; doi:10.1038/srep06256)
Supplement: Supplementary Information — Dataset 1 [file srep06256-s1.doc]

Supplementary Information

Electronic Coupling and Catalytic Effect on H_2_ Evolution of MoS_2_/Graphene Nanocatalyst

Ting Liao^1,2^*, Ziqi Sun^2^, Chenghua Sun^1,3^, Shixue Dou^2^, and Debra J. Searles^1,4^*

^1^AIBN Centre for Theoretical and Computational Molecular Science, University of Queensland, Brisbane, QLD 4072, Australia, ^2^Institute for Superconducting & Electronic Materials, University of Wollongong, Wollongong, NSW 2500, Australia, ^3^School of Chemistry, Monash University, Clayton, VIC 3800, Australia, ^4^School of Chemical and Molecular Biosciences, University of Queensland,
Brisbane, QLD 4072, Australia

We follow the approximated method proposed by Nørskov (J. Electrochem. Soc. 152, J23 (2005)) to estimate the difference in free energy for hydrogen binding to an isolated MoS_2_ nanolayer and and to MoS_2_@graphene as well. In supplementary figure 1, we plot the free energy of the hydrogen adsorbed state calculated as

Δ*G*_bond_=Δ*E*_bond_+Δ*E*_ZPE_-TΔ*S*_bond_

where Δ*E*_bond_ is the hydrogen bonding energy as plotted in Figure 3 and Δ*E*_ZPE_ is the difference in zero point energy between the hydrogen bonded states and the gas phase. We assumed that the vibrational entropy of bound hydrogen is small, hence the entropy of hydrogen bonding can be expressed as Δ*S*_bond_=-n/2S_H2_, where S_H2_ is the entropy of H_2_ in the gas phase at standard conditions. Δ*E*_ZPE_ are calculated to be 0.005 eV/supercell for the hydrogen adsorbed states which is much smaller than the -TΔ*S* change. Hence the free energy of the hydrogen adsorbed state was simply estimated as Δ*G*_bond_=Δ*E*_bond_+0.2 eV.


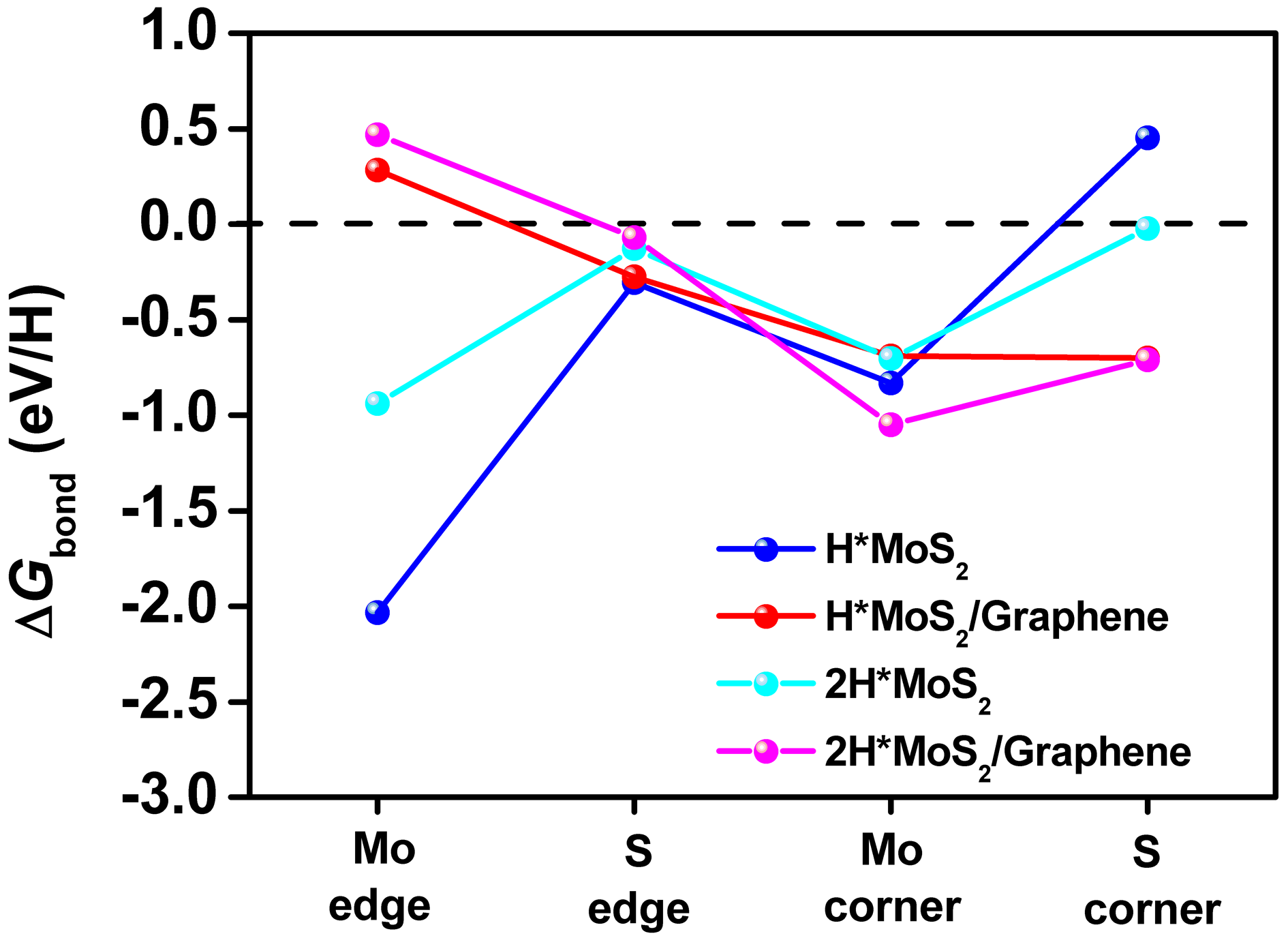


Figure 1. (a) The calculated hydrogen bonding free energy Δ*G*_bond_ (H and 2H) at the peripheral sites of MoS_2_/graphene nanocontact and free-standing MoS_2_ nanolayer.

**Table 1.** The calculated bonding energies (in eV) of sulphur atoms on the Mo edge sites of the MoS_2_/graphene nanocontact and free-standing MoS_2_ nanolayer.

|  | | |
| --- | --- | --- |
| Δ*E*_bond_(eV) | MoS_2_/Graphene | MoS_2_ |
| One-S-atom | -3.11 | -4.44 |
| Two-S-atom | -2.69 | -3.76 |
|  | | |

**Table 2.** The calculated bonding energies (in eV) of two hydrogen atoms on the Mo and S edge sites of the MoS_2_/graphene nanocontact and free-standing MoS_2_ nanolayer with van der Waals correction.

|  | | | |
| --- | --- | --- | --- |
| Δ*E*_bond_(eV) | | MoS_2_/Graphene | MoS_2_ |
| Mo-edge | 0.263 | | -1.143 |
| Mo-edge VdW | 0.232 | | -1.094 |
| S-edge | -0.276 | | -0.332 |
| S-edge VdW | -0.280 | | -0.339 |
|  | | | |
